# Human urine-derived stem cells in combination with polycaprolactone/gelatin nanofibrous membranes enhance wound healing by promoting angiogenesis

**DOI:** 10.1186/s12967-014-0274-2

**Published:** 2014-10-02

**Authors:** Yinxin Fu, Junjie Guan, Shangchun Guo, Fei Guo, Xin Niu, Qiang Liu, Changqing Zhang, Huarong Nie, Yang Wang

**Affiliations:** Institute of Microsurgery on Extremities, Shanghai Jiaotong University Affiliated Sixth People’s Hospital, Shanghai, 200233 China; Department of Clinical Laboratory, Pu’ ai Hospital Affiliated to Tongji Medical College, Huazhong University of Science and Technology, Wuhan, Hubei China; Jiangxi Origin Bio-TECH Co. LTD, Nanchang, 330006 China; Jiangxi Chuanqi pharmaceutical Co., LTD, Nanchang, 330039 China

**Keywords:** Human urine-derived stem cells (USCs), Angiogenesis, Endothelial cells, Polycaprolactone/gelatin (PCL/GT)

## Abstract

**Background:**

Despite advancements in wound healing techniques and devices, new treatments are needed to improve therapeutic outcomes. This study aimed to evaluate the potential use of a new biomaterial engineered from human urine-derived stem cells (USCs) and polycaprolactone/gelatin (PCL/GT) for wound healing.

**Methods:**

USCs were isolated from healthy individuals. To fabricate PCL/GT composite meshes, twin-nozzle electrospinning were used to spin the PCL and gelatin solutions in normal organic solvents. The morphologies and hydrophilicity properties of PCL/GT membranes were investigated. After USCs were seeded onto a PCL/GT, cell adhesion, morphology, proliferation, and cytotoxicity were examined. Then, USCs were seeded on a PCL/GT blend nanofibrous membrane and transplanted into rabbit full-thickness skin defects for wound repair. Finally, the effect of USCs condition medium on proliferation, migration, and tube formation of human umbilical vein endothelial cells (HUVECs) were performed in vitro.

**Results:**

USCs were successfully isolated from urine samples and expressed specific mesenchymal stem cells markers and could differentiate into osteoblasts, adipocytes, and chondrocytes. PCL/GT membrane has suitable mechanical properties similar with skin tissue and has good biocompatibility. USCs-PCL/GT significantly enhanced the healing of full-thickness skin wounds in rabbits compared to wounds treated with PCL/GT membrane alone or untreated wounds. USCs-PCL/GT-treated wounds closed much faster, with increased re-epithelialization, collagen formation, and angiogenesis. Moreover, USCs could secrete VEGF and TGF-β1, and USC-conditioned medium enhanced the migration, proliferation, and tube formation of endothelial cells.

**Conclusion:**

USCs in combination with PCL/GT significantly prompted the healing of full-thickness skin wounds in rabbits. USCs based therapy provides a novel strategy for accelerating wound closure and promoting angiogenesis.

## Introduction

Autologous, allograft, and xenograft skin transplants are often employed to treat large areas of skin damage caused by wounds, ulcers, burns, or inflammation. However, autologous skin grafts are limited by their sources and by defects at donor sites, and allograft and xenograft skin grafts are limited by the risk of immune rejection or infectious disease; thus, meeting the needs of clinical applications is difficult [1-3]. Tissue engineered skin has recently become a promising strategy for treating large skin defects and skin ulcers that are difficult to heal [4-6], and stem cells have shown unique advantages for the construction of engineered skin tissue. Adipose-derived mesenchymal stem cells (ASCs) seeded in sodium carboxymethyl cellulose (CMC) promoted wound healing in rats [7]. ASCs seeded in a composite elastic protein electrostatic nanofiber also effectively promoted mouse wound healing [8]. However, the acquisition of adult stem cells is often limited by the source and by invasive routes of collection and embryonic stem cells or induced pluripotent stem cells have issues with respect to ethics, immune rejection, tumorigenicity, and other areas. Therefore, the identification of a new source of autologous stem cells that is easy to obtain via a non-invasive route is imperative.

Recently, stem-like cells have been extracted from adult urine and named urine derived stem cells (USCs) [9-12]. These cells exhibit stem cell markers similar to those of ASCs and the ability to differentiate into osteocytes, chondrocytes, adipocytes, and endothelial cells [12]. USCs have been studied as an alternative to cell therapy and tissue engineering for urological applications during the treatment of stress urinary incontinence and vesicoureteral reflux and during bladder and urethra tissue engineering [10,11,13] and nerve damage repair [14]. However, the application of USCs to skin tissue engineering has not been reported.

The identification of appropriate scaffold materials for skin tissue engineering is a challenge. The application of stem cells in combination with biomaterials plays an increasingly prominent role in tissue engineering and regenerative medicine [15]. An appropriate tissue scaffold should confer mechanical properties and good biocompatibility for wound healing [16]. A large fraction of electrospun nanofibrous mesh exists in the form of interconnected porosity [17-19]. Compared to regular fibers, these nanofibers have a higher-specific surface area, a higher aspect ratio, and higher porosity; these characteristics give the mats strong absorbability, good filtration, effective seclusion, and high conglutinating quality, providing good accommodation for the cells and easy passage for nutrient intake and metabolic waste exchange. A large number of electrospun meshes have been used for wound regeneration, particularly polycaprolactone (PCL)-based mats, which are regarded as safe by the FDA, with good mechanical properties [20,21]. However, slow degradation and poor hydrophilicity are problems for PCL applications in tissue engineering [22]. Therefore, a large number of PCL-based composite meshes, particularly PCL/gelatin (PCL/GT) composite membranes, were developed to improve the adaptability of these meshes [23,24].

PCL/GT mesh is typically obtained by co-spinning solutions of PCL and gelatin in expensive fluoro-substituted solvents [25,26]. Here, we used twin-nozzle electrospinning to spin the PCL and gelatin solutions in normal organic solvents to fabricate PCL/gelatin composite meshes. We then investigated the benefits of USCs in combination with PCL/GT nanofibrous membranes for wound healing in a rabbit full-thickness skin excision wound model. Finally, we tested the effects of USCs condition medium on proliferation, migration, and tube formation of HUVECs.

## Materials and methods

### Isolation and purification of USCs

This study was performed in accordance with the principles of the Helsinki Declaration and was approved by the Ethical Review Board of Shanghai Six People’s Hospital Affiliated with Jiaotong University. Human urine samples were obtained from eight healthy men with a mean age of 25 years (age range 20–28 years). Written informed consent was obtained from all study participants.

To isolate USCs, penicillin and streptomycin were added to the fresh urine samples (200 ml) at the recommended concentrations to minimize contamination. After the urine sample was centrifuged for 10 minutes at 400 × g and room temperature, the supernatant was discarded and the sediment was washed twice with phosphate-buffered saline (PBS). The resulting sediment was re-suspended in Dulbecco’s Modified Eagle Medium (DMEM) supplemented with 2% (vol/vol) fetal bovine serum (FBS) (Gibco, USA), 10 ng/ml of human epidermal growth factor (hEGF), 2 ng/ml of platelet-derived growth factor (PDGF), 1 ng/ml of transforming growth factor-β (TGF-β), 2 ng/ml of basic fibroblast growth factor (bFGF), 0.5 μM hydrocortisone, 25 μg/ml of insulin, 20 μg/ml of transferrin, 549 ng/ml of epinephrine, 50 ng/ml of triiodothyronine (T3), L-glu and antibiotics. The cell suspension was plated into gelatin-coated 24-well plates and incubated at 37°C in a humidified atmosphere with 5% CO_2_. The medium was changed after 7 days, and the non-adherent cells were removed by thoroughly washing with PBS. Colonies derived from single cells were marked. The culture medium was refreshed twice per week. The cells were passaged using 0.25% trypsin when they reached approximately 80% confluence.

### Multipotent differentiation potential of USCs

#### Osteogenic induction

Cultured USCs at passage 4 were grown to 80% confluence and subsequently incubated with osteogenic induction medium (Gibco, USA). After 21 days of induction, the cells were stained with Alizarin Red S to detect the deposition of calcified matrix.

#### Adipogenic induction

The cultured USCs were grown to 80% confluence and incubated with adipogenesis medium (Gibco, USA). The culture medium was changed every 3 days. After 14 days of induction, the cells were fixed in 4% paraformaldehyde (PFA) for 30 min at RT and stained with Oil Red O for 10 min.

#### Chondrogenic induction

Chondrogenic differentiation of USCs was performed using a pellet culture technique. Briefly, 1 × 10^6^ cells were pelleted by centrifugation in a 15-ml centrifuge tube. Chondrogenic medium (Gibco, USA) was gently added to the pellet. After 4 weeks of induction, the pellets were fixed in 4% PFA and embedded in an optimum cutting temperature (OCT) compound. The 10-μm cryosections were stained with Toluidine blue. All of the stained cells were observed under an optical microscope (Leica, Germany).

### Flow cytometry analysis

Passage 4 USCs were incubated with 3% BSA for 30 minutes to block nonspecific antigens. The cells were then incubated with the following monoclonal antibodies (Becton Dickinson): CD29-PE, CD146-FITC, CD73-PE, CD90-FITC, CD34-APC and HLA-DR-PE. The cells were washed to remove unbound antibody. Surface antigens were analyzed using a Guava easyCyte™ (Millipore, USA).

### Preparation of PCL/GT membranes

PCL/GT membranes were prepared as previously described [27]. PCL was dissolved at 10% (wt/vol) in mixtures of 7:1 (vol/vol) dichloroform and dimethylbenzene. Gelatin was dissolved at 20% (wt/vol) in mixtures of 2:1 (vol/vol) formic acid and ethyl acetate. A twin-spinneret electrospinning technique was employed to produce PCL and gelatin composite fibers using the parameters described below. We dispensed the PCL solution at a flow rate of 150 μl min^−1^ and the gelatin solution at a flow rate of 60 μl min^−1^ through a 7-gauge stainless steel dispensing needle that was clamped to +15 kV using a high voltage generator. The collector was placed 12 cm from the tip of the needle horizontally and set to 500 r.p.m. After 1.5 mL of the gelatin solution and 3 mL of the PCL solution were electrospun simultaneously, the electrospun hybrid meshes were removed from the collector and lyophilized (LGJ-10C, Four-Ring Science Instrument Plant Beijing Co., Beijing, China) for at least 24 h prior to imaging or use in biological assays.

### Characterization of PCL/GT membranes

The morphologies of the electrospun membranes were observed using a scanning electron microscope (SEM) (FEIQuanta, 200SEM) at an accelerating voltage of 30 kV. Each sample was sputter-coated with platinum for analysis. The FTIR spectra were recorded on an infrared spectrometer (IR, Nioclet 5700) at a 4 cm^−1^ resolution, with 32 scans in the range of 4,000 to 400 cm^−1^. Hydrophilicity properties were determined using a contact angle goniometer (KROSSDSA100, Germany) at room temperature with 2-μL water droplets. The reported contact angle value represents an average of at least five independent determinations at different sites.

The scaffolds were cut into small pieces with sizes of 9 cm^2^. The *in vitro* degradation experiment was performed in 50 mL of phosphate-buffered saline (PBS) (pH = 7.4) at 37°C for different time intervals. The degradation rate was evaluated using the observed weight changes before and after immersion, according to equation (1).

1 $$ \mathrm{Weight}\ \mathrm{loss}\left(\%\right)=\left(\mathrm{W}\mathrm{d}-\mathrm{W}\mathrm{r}\right)/\mathrm{W}\mathrm{d}\times 100\% $$

Wr represents the remaining weight (after drying) of the membranes after immersion in PBS, and Wd represents the original weight of the same membranes prior to immersion.

The mechanical properties of 17.8 mm × 26.6 mm membrane specimens with thicknesses of 220 μm were determined using a material testing system with electronic data evaluation (Zwick/Roell, BZ2.5/TS1S, Germany).

### Cell morphology, viability and proliferation in PCL/GT Membranes

Membranes seeded with USCs were rinsed in 0.1 M phosphate buffer (pH 7.2) and fixed with 2.5% glutaraldehyde in 0.1 M phosphate buffer for 2 hours at 4°C. To prepare for SEM, the samples were dehydrated with ethanol and subsequently placed in pure tert-butanol for 30 minutes prior to vacuum drying overnight. After sputter-coating with platinum, the morphologies of the cells and the scaffold and cell attachment to the PCL/GT membranes were analyzed using a SEM (FEIQuanta 200).

The viability of the cells on the PCL/GT membranes was determined using a live/dead cell staining kit (ScienCell, USA). Green fluorescence, which occurs due to the reaction of calcein with intracellular esterase, indicated live cells; while red fluorescence occurs due to the binding of ethidium homodimers to nucleic acids, indicated dead cells. The USCs were seeded on PCL/GT membranes that were commensurate with the size of 24-well plates at a density of 2 × 10^4^ cells/well in 300 μL of medium. The same amount of culture medium was added to a well containing a PCL/GT membrane to serve as a control.

Cell proliferation was assessed using a Cell Count-8 kit (CCK-8) (Dojindo Molecular Technologies, Inc., Kumamoto, Japan) according to the manufacturer’s protocol. The plates were then evaluated at 450 nm using an enzyme-linked immunosorbent assay instrument. The optical density values were calculated from triplicate samples of each group, and the experiment was repeated four times.

### USCs for skin tissue engineering in rabbits

All in vivo experimental procedures were approved by the Animal Research Committees of Shanghai Six People’s Hospital. The rabbits were housed individually in standard cages in a room with controlled temperature and light in the Facility of the Animal Research Centre at Shanghai Six People’s Hospital. Nine male New Zealand White rabbits (3–3.5 Kg) were used for the transplantation of USCs in combination with PCL/GT membranes and injected with the immunosuppressant cyclosporine A at a dose of 5 mg/kg per day. The full-thickness skin excision wound animal model was established as previously described [28]. On each rabbit, a total of four full-thickness skin wounds (2 cm × 2 cm) were made dorsally, with two wounds on each side of the paravertebra. The rabbits were treated with PCL/GT membranes or USCs-PCL/GT membranes, with an untreated group as a control. All animals were healthy after surgery without weight loss and were alive at the end of the experimental period. The adhesive was tested on the skin of rabbits prior to this experiment, and no skin irritation or allergic reaction was observed. The animals were killed 7 or 14 days after surgery. The wound area and the surrounding outer skin were collected for histological examination.

### Wound analysis

Digital photographs of the wounds were taken on day 14 after surgery. The time to wound closure was defined as the time at which the wound bed was completely re-epithelialized and filled with new tissue. For whole skin mounts, the entire wound and the surrounding skin were placed on a plastic dish with the dermis side down and photographed immediately. The wound area was measured group-blindly by tracing the wound margin and calculated using the Image-Pro Plus 6 program (Media Cybernetics, Rockville, MD). The percentage of wound closure was calculated as follows: [(Area of original wound - Area of actual wound)/Area of original wound] × 100%.

### Histologic and immunocytochemical analysis

The tissue specimens were fixed in 4% PFA for 24 hours and embedded in OCT (ThermoFisher, Waltham, MA). Six-micron sections were stained with hematoxylin and eosin (H&E). Histological evaluation was performed based on previous reports[29,30], including re-epithelialization and regeneration, collagen formation, and angiogenesis. Masson’s trichrome staining was employed to visualize collagen fibers. All sections were analyzed by two pathologists in a blinded manner using light microscopy (Leica).

For immunofluorescence, the tissue sections were pre-incubated with sodium borohydride (1 mg/ml in PBS) to reduce autofluorescence. The area of the epidermis was measured by staining keratinocytes with an antibody against epidermal pan-cytokeratin (Abcam, 1:100). After immunofluorescent staining of endothelial cells with an anti-CD31 antibody (Abcam, 1:50), the microvessel density was assessed morphometrically by examining three fields per wound section [31,32]. A secondary IgG antibody conjugated to Alexa Fluor 594 (Jackson, 1:200) was used for visualization. The nucleus was stained with 4′, 6-diamidino-2-phenylindole (DAPI). Isotype control antibodies were used as negative controls. All stained cells were observed under a fluorescence microscope (Leica).

The percentages of keratinocytes and collagen areas were determined by counting in five random fields per section between wound edges using Image-Pro Plus 6.

### Enzyme-linked immunosorbent assay

To measure angiogenic trophic factors secreted by the USCs, 1 × 10^5^ USCs at passage 4 were seeded in 6-well plates and incubated with serum-free DMEM under normal conditions (i.e., 5% CO_2_ and 37°C). The culture medium of the USCs was replaced every 2 days, and the culture supernatant was aspirated after 48 h for the measurement of VEGF and TGF-β1 using an enzyme-linked immunosorbent assay (ELISA) according to the manufacturer’s instructions (R&D, Systems, USA).

### Endothelial cell migration and proliferation assay

RTCA (real-time cell analyzer) migration assays measure the effects of perturbations in a label-free, real-time setting. As cells migrate from the coated upper chamber through the membrane and into the bottom chamber in response to a chemo-attractant, they contact and adhere to the electronic sensors on the underside of the membrane, resulting in an increase in the electrical impedance. Increasing impedance correlates with increasing numbers of migrated cells on the membrane [33].

HUVECs were obtained from ScienCell (Carlsbad, CA, USA). The cells were cultured in Medium M200 using low serum growth supplementation (LSGS) kits (Cascade Biologics, USA) at 37°C in a humidified 5% CO_2_ atmosphere. HUVECs were used at passage number 4 in this study. For RTCA migration assays, 4 × 10^4^ HUVECs were seeded into the upper well of each transwell chamber (8 μm pore size). To observe the effects of USCs-conditioned medium (i.e., culture supernatant of USCs after 3 days) on the migration of HUVECs,M200 medium containing 25% USCs-conditioned medium or USCs-medium was tested to determine the optimal concentration. USCs-CM (i.e., M200 medium containing 25% USCs-conditioned medium), USCs-M (i.e., USCs-medium), and Ctrl (i.e., M200 medium) (300 *μ*l) containing 5% FBS were placed in the lower compartment of the chemotaxis chamber as a source of chemo-attractants. The cells were incubated for 16 h at 37°C in a 5% CO_2_ atmosphere. Cells that had migrated to the lower surface of the membrane were measured using an RTCA analyzer system (Roche, USA).

A wound healing assay was performed as a supplement to migration ability. Briefly, 2 × 10^5^ cells were plated in 12-well plates and grown to confluence. Two parallel scratches were made in each dish using a 200 μL pipette tip, and the scratch width was measured as the baseline [34]. The wound width was measured using Image J software (National Institutes of Health, Bethesda, MD) by mimicking an exclusion zone assay. USCs-M and USCs-CM were added for 24 h. The width of the scratches was visualized using a microscope (Leica, Germany).

Proliferation was evaluated using a CCK-8 kit (Dojindo, Japan) according to the manufacturer’s instructions. Briefly, HUVECs were seeded into 96-well plates in 100 μl of culture medium (i.e., Ctrl, USCs-M or USCs-CM) at a density of 5,000 cells per well. After 24, 48, 72, and 96 h, 10 μl of CCK-8 solution were added to each well and the plates were incubated at 37°C for 3 h. At the end of the incubation, the absorbance at 450 nm was measured using a microplate reader (Bio-Rad Laboratories Co. USA). All experiments were performed in triplicate and repeated at least 3 independent times.

### Endothelial cell network formation assay

Human umbilical vein endothelial cells (HUVECs) (2 × 10^4^ cells per well) were suspended in 100 μl of USCs-M or USCs-CM and seeded onto Matrigel (BD Biosciences, USA)-coated 96-well plates. After incubation at 37°C with 5% CO_2_ for 6 hours, images were captured. Four random fields were evaluated for each well.

### Statistical analysis

The data are expressed as the mean ± SD. Intergroup comparisons were performed using ANOVA followed by Tukey post hoc analysis. All data analysis was performed using SPSS 17.0 (SPSS Inc., Chicago, IL, USA). P values less than 0.05 (p < 0.05) were considered statistically significant.

## Results

### Characterization of USCs

**Figure 1:**
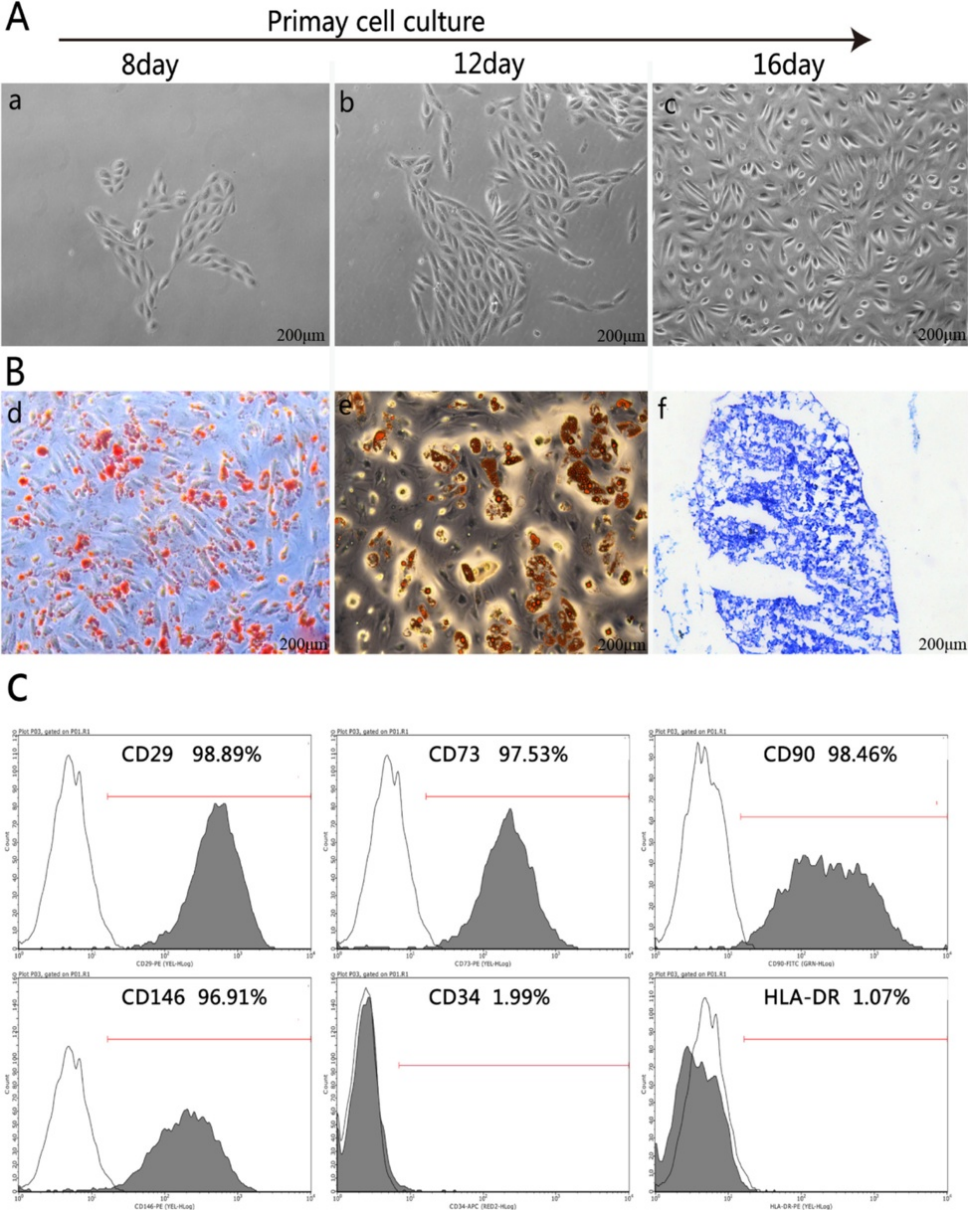
**The characteristics of USCs. A**. The morphology and growth of USCs; **B**. Differentiation of USCs into osteoblasts (d), adipocytes (e), and chondrocytes (f); **C**. USCs were characterized by flow cytometry using the surface markers CD29, CD73, CD90, CD146, CD34 and HLA-DR. Scale bar = 200 μm.

Cell colonies were observed in the USCs cultured plates approximately 8 to 12 days after initial plating. Most of the adherent cells exhibited a fibroblast-like morphology (Figure 1A). After 16 days, the fibroblast-like cells retained a robust proliferation capability and reached 80%-90% confluence (Figure 1A). No microorganism contamination was observed in any of the culture plates. When cultured in osteogenic, adipogenic, or chondrogenic medium, the USCs differentiated into osteoblasts (Figure 1B:d), adipocytes (Figure 1B:e), or chondrocytes (Figure 1B:f), respectively. Flow cytometry analyses demonstrated that the USCs were positive for CD29, CD73, CD146 and CD90 antigen and negative for CD34 and HLA-DR (Figure 1C). Thus, the characterization of USCs meets the criteria for defining multipotent MSCs [35].

### Characterization of the PCL/GT membranes

**Figure 2:**
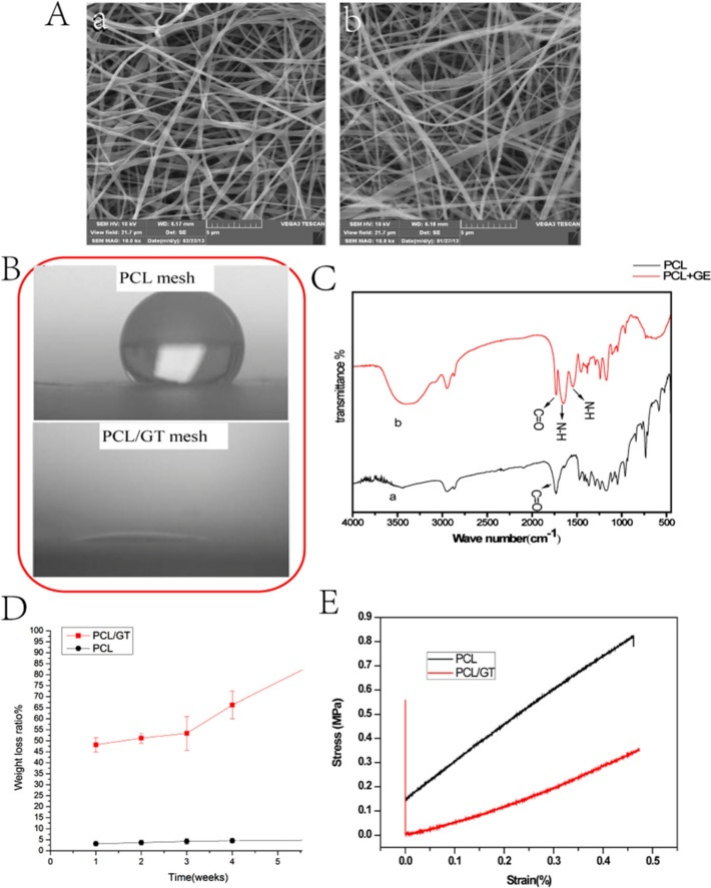
**The characteristics of PCL/GT.** SEM images of electrospun PCL/GT (**A**: a) and PCL (**A**: b) membranes; **(B)** images of water droplets on PCL and PCL/GT scaffolds; **(C)** FTIR spectra of electrospun membranes; **(D)** Degradation behavior of PCL/GT composite mesh; **(E)** Stress–strain curves of the fibrous membranes. Scale bar = 5 μm.

Figure 2A (a,b) shows the morphology of the electrospun PCL and PCL/GT composite fibrous membranes. The morphologies of PCL/GT (Figure 2A:b) and PCL (Figure 2A:a) exhibited randomly oriented non-woven fibers with an open porosity and interconnected pores. No differences in average fiber size, the distribution of fiber sizes or pore size were observed between the PCL-GT fibrous mesh and the PCL membranes. Figure 2C shows the infrared spectra of the PCL and PCL/GT composite membranes. The strong absorption band of the PCL/GT mesh at 3,400–3,500 cm^−1^ can be attributed to the overlapping N–H and O–H stretching vibrations of GT. The absorption of the N–H deformation at 1,600–1,650 cm − 1 and 1,500–1,550 cm^−1^ are also assigned to GT. The absorption peak at 1,700–1,760 cm^−1^ occurs due to C = O groups. For PCL membranes, all of the observed absorption bands resulted from the chemical structure of PCL.

Contact angles of water droplets have been used to evaluate the wettability of proposed membranes. As shown in Figure 2B, the PCL membranes exhibited hydrophobic characteristics, with water contact values higher than 90°. However, after the codeposition of GT nanofibers, the static contact angle of the composite mesh decreased to nearly 0 from the value observed for the PCL membranes (109 ± 0.4)due to the existence of a large number of polar groups along the GT molecular chains.

As shown in Figure 2D, the PCL/GT membranes lost ∼ 50% of their initial weight after 1 week of degradation in PBS. Moreover, this weight loss increased sharply after 6 weeks. The observed 50% loss of the composite scaffold weight results completely from the dissolution of the GT component in the PCL/GT mesh. This result suggests that GT could promote the degradation of PCL.

The mechanical strength of scaffolds is an important factor that must be considered in the context of applications in skin tissue engineering. The typical strain–stress curves of the two types of scaffolds are presented in Figure 2E. The Young’s moduli of the PCL scaffolds and the PCL/GT scaffolds are 1.43 MPa and 0.86 MPa, respectively. The Young’s moduli of the volar forearm, the dorsal forearm, and the palm are 101.180, 68.678, and 24.910 kPa, respectively [36]. This result demonstrates that the mechanical properties of PCL/GT fibrous membranes are comparable to those of human skin tissue.

### Biocompatibility of PCL/GT membranes in vitro

**Figure 3:**
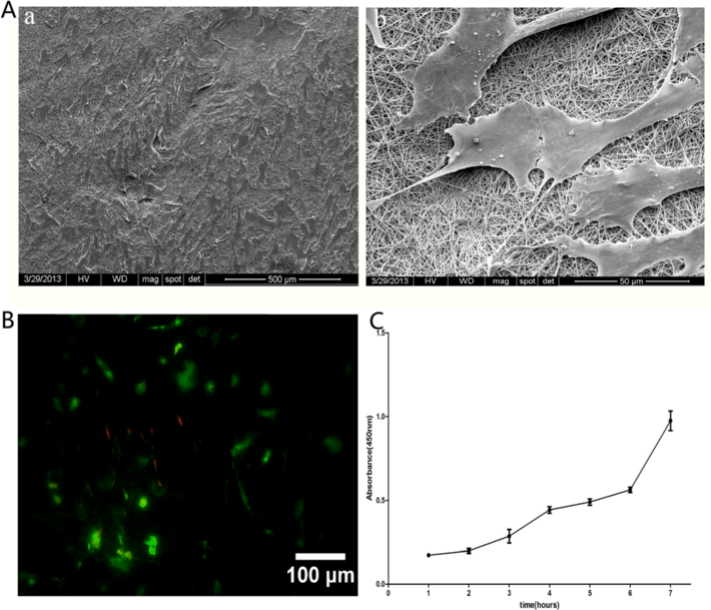
**The biocompatibility of PCL/GT membranes. A**. SEM images of USCs on PCL/GT on day 7, a: low magnification, b: high magnification; **B**. The viability of USCs, as analyzed using a live/dead assay in which calcein green indicates live cells; **C**. USCs showed robust proliferation on PCL/GT. Scale bar A (a) = 500 μm; A (b) = 50 μm; B = 100 μm.

The USCs were seeded onto PCL/GT membranes. After seeding the cells onto PCL/GT, the USCs adhered to and spread on the membrane 1 day after seeding. SEM analyses revealed that the cells proliferated continuously on the membrane and reached approximately 85% confluency after 7 days of culture (Figure 3A). Seven days after the USCs were seeded onto PCL/GT membranes, a live/dead assay demonstrated that the USCs seeded in the 3D PCL/GT membranes were still alive (Figure 3B). These results were confirmed using CCK-8 analysis. Increases in optical density were observed with increasing culture time (Figure 3C). These data demonstrated that PCL/GT membranes possess good biocompatibility.

### Gross observation of wounds

**Figure 4:**
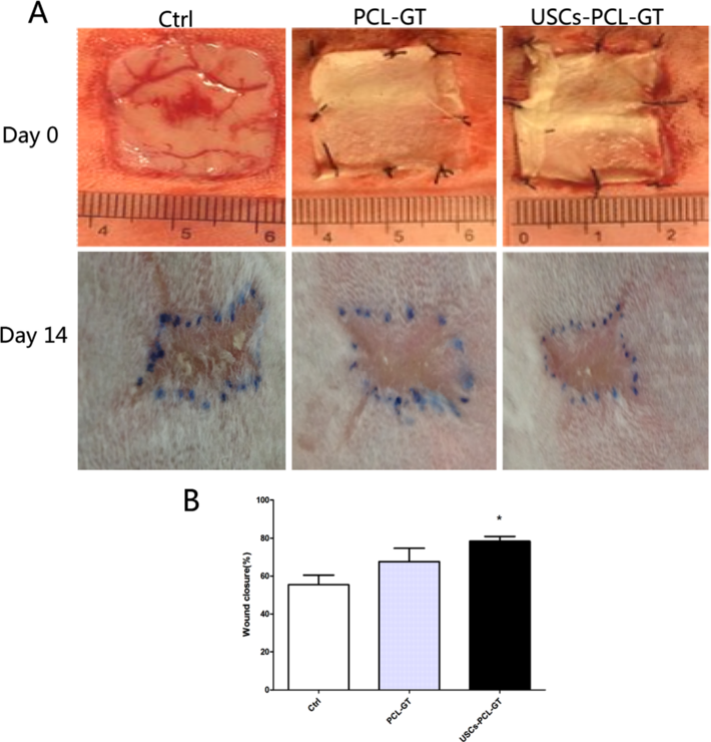
**The wound areas were analyzed on day 0 and day 14 post-surgery in the control, PCL/GT, and USCs-PCL/GT groups. (A)** Gross appearances of wounds on day 0 and day 14. **(B)** Significantly faster wound closure was observed in the USCs-PCL/GT group (*P < 0.05 compared to the PCL/GT group or the Ctrl group).

All wounds were examined and recorded on days 7 and 14 post-surgery. After 7 days, all cotton dressings were removed. All wounds in the PCL/GT membrane-treated groups were moist. However, the wounds of the control groups were dry. These results demonstrate that the scaffolds (i.e., the PCL/GT membranes and the USCs-PCL/GT membranes) are able to keep wounds moist. By day 14, wound healing was observed in all groups, including the treatment groups and the control groups (Figure 4A). The wounds treated with USCs-PCL/GT exhibited significantly improved wound contraction. On day 21, the wounds of all groups were nearly completely epithelialized. The wounds that received USCs- PCL/GT closed faster than those that received PCL/GT or remained untreated (Figure 4B).

### USCs-PCL/GT membranes enhance wound healing

**Figure 5:**
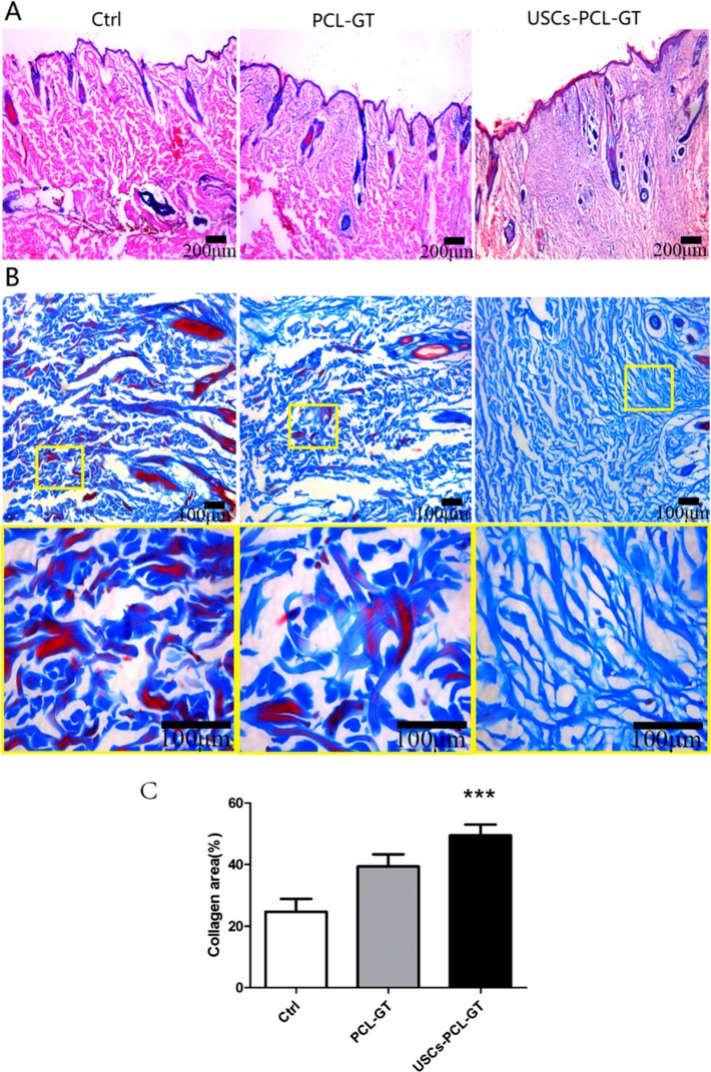
**Histological analysis of rabbit wounds on day 14. (A)** Wound histological images (H&E stain); **(B)** Masson’s trichrome staining, the bottom shows an enlarged view of the region inside the box; **(C)** Areas of collagen were quantified in day 14 wounds (Image Pro Plus 6). Significantly greater collagen area was observed in the USCs-PCL/GT group than in the control or PCL/GT groups (***P < 0.01). Scale bar A = 200 μm, B = 100 μm.

**Figure 6:**
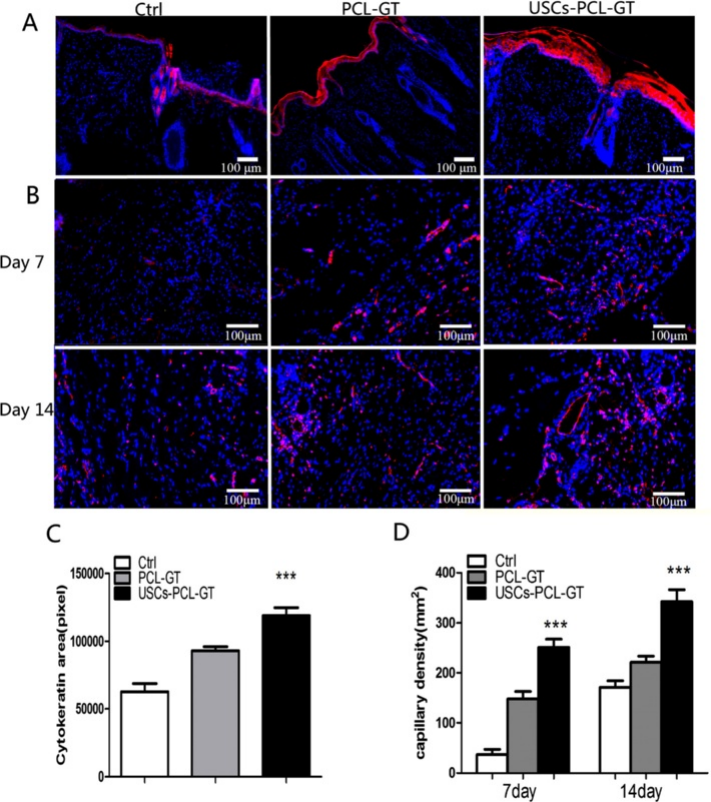
**Tissue sections were immunostained with anti-pan-cytokeratin (A) and anti-CD31 antibodies (B).** Nuclei (blue) were stained with DAPI. **(C)** Significantly greater cytokeratin area was observed in the USCs-PCL/GT group (***P < 0.01 compared to the control group, *P < 0.05 compared to the PCL/GT group). **(D)** Significantly increased microvessel density was observed in the USCs-PCL/GT group (***P < 0.01 compared to the control and PCL/GT groups). Scale bar = 100 μm.

A histological evaluation of the rabbit wounds after 14 days revealed enhanced re-epithelialization in the USCs-PCL/GT treated wounds compared to the PCL/GT-treated wounds or the untreated wounds (Figure 5A). The granulation tissue in the USCs-PCL/GT-treated wounds appeared to be thicker and larger than that of the PCL/GT-treated wounds or the untreated wounds (Figure 5A). After 14 days, the collagen content was significantly higher in the USCs-PCL/GT group than in the control and PCL-GT groups (Figure 5B, C). Moreover, collagen was well organized and skin appendages, such as hair follicles and sebaceous glands, were found in the USCs-PCL/GT-treated wounds. Additionally, immunohistological staining of tissue sections for cytokeratin demonstrated that USCs-PCL/GT-treated wounds were better epithelialized than those of the other groups (Figure 6A, C).

### USCs -PCL/GT membranes enhance angiogenesis in the wound tissue

Immunohistological staining of tissue sections for the endothelial marker CD31 (Figure 6B) revealed increased microvessel density in USCs-PCL/GT-treated wounds after 7 and 14 days compared to control or PCL/GT-treated wounds. The microvessel density in the wounds on day 14 was assessed morphometrically after immunohistochemical staining for CD31. The microvessel density was significantly higher in USCs-PCL/GT-treated wounds than in control or PCL/GT-treated wounds (Figure 6B, D).

### USCs secrete angiogenic trophic factors and promote the proliferation, migration, and tube formation of HUVECs

**Figure 7:**
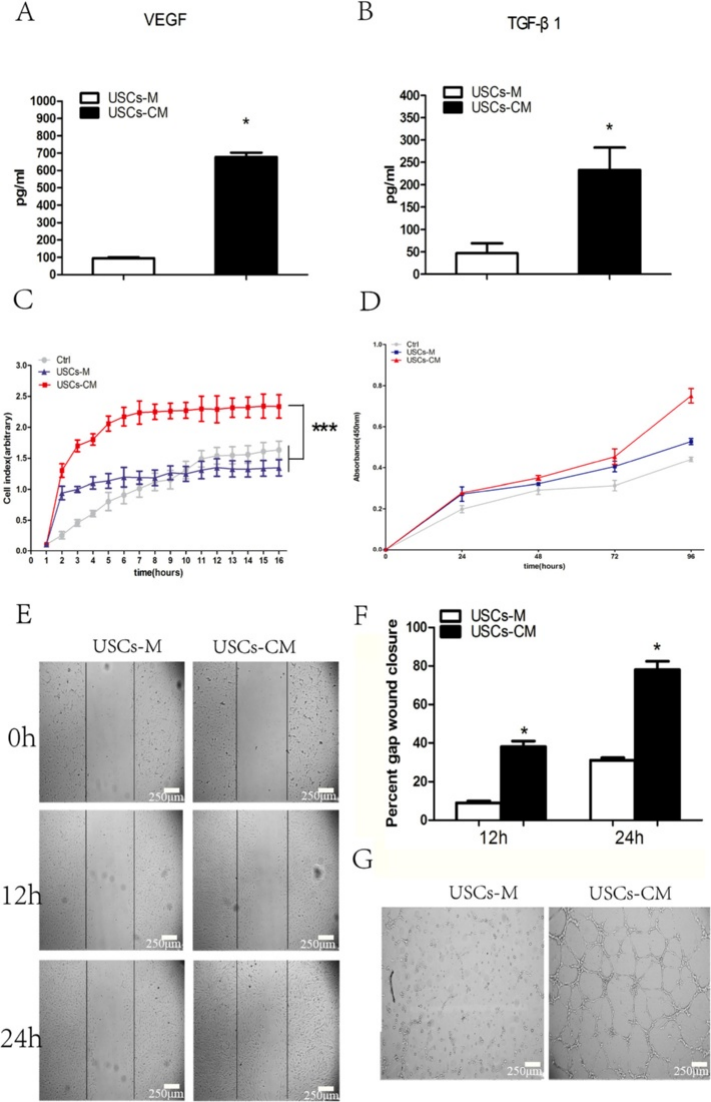
**The ELISA assay for USCs-CM and the effect of USCs-CM on HUVECs.** The concentration of VEGF **(A)** and TGF-β **(B)** in USCs-M and USCs-CM was measured by ELISA. **(C)** Real-Time Cell Analyzer showed USCs-CM significantly enhanced the migration of HUVECs. **(D)** CCK-8 assay showed USCs-CM significantly promoted HUVECs proliferation after 96 h incubation. **(E** and **F)** The scratch experiments demonstrated that USCs-CM increased the motility of HUVECs. **(G)** Large numbers of tube were formed in USCs-CM-cultured HUVECs, while no tube was formed in USCs-M-cultured HUVECs. *** P < 0.01 compared to the control or USCs-M groups at 16 h; *P < 0.05 compared to USCs-M groups. Scale bar = 250 μm.

To further delineate the mechanisms which angiogenesis is increased by USCs, the VEGF and TGF-β1 levels in the USCs culture supernatants were tested and found to be significantly higher than those of the controls (Figure 7A, B). We further observed the effects of USCs-conditioned medium (USCs-CM) on the proliferation, migration, and tube formation of human umbilical vein endothelial cells. USCs-CM significantly increased HUVEC proliferation compared to the controls (P < 0.05) (Figure 7D). The results of RTCA experiments demonstrated that USCs-CM significantly enhanced the motility of HUVECs (P < 0.01) (Figure 7C); this finding was confirmed by the results of scratch experiments. At 24 h after scratch initiation, USCs-CM-treated HUVECs covered nearly the whole scratch mark, while USCs-M left significant empty space in the scratch (P <0.05) (Figure 7E, F). After 6 h of culture on Matrigel, no tube was formed by USCs-M, while USCs-CM-cultured HUVECs formed many tubes with obvious structures (Figure 7G), indicating that paracrine factors in the USCs culture supernatant promoted angiogenesis.

## Discussion

The therapeutic approaches for extensive full-thickness skin defects that result from injuries, burns, other types of trauma, and non-healing ulcers remain very limited [37,38]; thus, innovative strategies for improving wound healing and regeneration are needed. Recently, tissue engineering skin has become a potential strategy for improving wound healing. In the present study, we demonstrated for the first time that the transplantation of biomaterials engineered by incorporating USCs into PCL/GT nanofibrous membranes enhanced wound healing in a rabbit full-thickness skin wound model. In addition, the observed protective effect was likely mediated by USCs via a paracrine mechanism that affects angiogenesis, as we found that USCs-conditioned medium could enhance endothelial cell migration, proliferation and tube formation.

Bone marrow-derived mesenchymal stem cells (BMSCs) promote angiogenesis and improve cutaneous wound healing [39,40]. However, the source of BMSCs is limited and the procedure for the isolation of these cells is invasive. Recently, USCs that can be harvested from voided urine via a non-invasive procedure have been reported to possess a high proliferative capacity and characteristics of stem cells and to exhibit potential for use in urinary tissue engineering [12,41,42]. Our previous study also found that USCs have multilineage differentiation potential and possess biological characteristics similar to ASCs [14]. Moreover, we found that USCs can differentiate into neuron-like cells in the rat brain, indicating that USCs are a promising cell source for tissue engineering and regenerative medicine [14]. The present data further demonstrated that USCs are beneficial for angiogenesis and can be used in skin tissue engineering.

As pre-clinical research investigating the cutaneous wound healing benefits of MSCs becomes more common, the majority of studies have investigated the direct injection of cell suspensions around the wound. Cells injected directly into the body undergo cell death rapidly [43]. Biomaterials may support cell viability and enhance therapeutic efficacy. PCL is a polyester that is widely used as a tissue engineering scaffold material due to its well-documented biocompatibility. However, the fact that PCL degrades much slower than other known biodegradable polymers limits the application of this polymer in vivo. The complete biodegradation of PCL in vivo typically takes more than 1 year due to the hydrophobicity and crystallinity of this polymer. In addition, for in vivo applications, biomaterials should be hydrophilic for improved biocompatibility; however, the hydrophilicity of PCL is very limited. In the present study, we used twin-nozzle electrospinning of PCL and gelatin solutions in normal organic solvents to fabricate PCL/gelatin composite meshes. The results demonstrate that the weight loss of the PCL/GT mesh may be attributed to the local environment of GT degradation, which favors the degradation of the PCL component. Importantly, this result may offer a new solution to the slow degradation of PCL in vivo. These results also demonstrate that the introduction of gelatin greatly improves the hydrophilicity and mechanical properties of PCL/GT fibrous membranes, achieving values comparable to those of human skin tissue. Together, our results demonstrate that PCL/GT nanofibrous membranes exhibit good biocompatibility and sufficient mechanical strength and can sustain cellular viability. These membranes can also provide an extracellular matrix (ECM) for wounds to promote cellular adhesion and proliferation, making them suitable scaffolds for skin engineering.

Neovascularization is a crucial step in the wound healing process [38,44]. The formation of new blood vessels is necessary to sustain the newly formed granulation tissue and promote the survival of keratinocytes. Our data demonstrated that USCs-PCL/GT-treated wounds increased the capillary density on days 7 and day 14, suggesting that USCs promoted angiogenesis. Paracrine factors from BM-MSCs have been reported to recruit macrophages and endothelial lineage cells into the wound and enhance wound healing [45]. The present study represents the first report that USCs can secrete angiogenic trophic factors, including VEGF and TGF-β1, and that USCs-conditioned medium can significantly promote endothelial tube formation and endothelial cell migration and proliferation, indicating that paracrine factors released by USCs play a key role in angiogenesis by stimulating endothelial cell proliferation, migration and organization into tubules.

Cyclosporin A is widely used in organ and tissue transplantation to prevent the immune rejection. It’s has demonstrated that Cyclosporin A improve the survival rate of graft cells [46]. In this study, consistent with prior reports, the risk of immune rejection was significantly lower with the use of Cyclosporin A. To examine whether Cyclosporin had a side effect on skin wound healing, we injected Cyclosporin A in some rabbits in the Control groups. The results showed there is no difference in wound healing between the injected and un-injected rabbits, which confirmed the application of Cyclosporin A had no side effect on wound healing.

## Conclusion

This study reported the beneficial effect of USCs on cutaneous regeneration and wound healing via systemic paracrine effects that promote angiogenesis. We believe that USCs in combination with PCL/GT nanofibrous membranes represent a novel therapeutic approach to the treatment of skin defects.

## Abbreviations

USCs: Human urine-derived stem cells
PCL/GT: Polycaprolactone/gelatin
HUVECs: Human umbilical vein endothelial cells
ASCs: Adipose-derived mesenchymal stem cells
CMC: Carboxymethyl cellulose
PBS: Phosphate-buffered saline
DMEM: Dulbecco’s Modified Eagle Medium
FBS: Fetal bovine serum
hEGF: Human epidermal growth factor
PDGF: Platelet-derived growth factor
TGF-β: Transforming growth factor-β
bFGF: Basic fibroblast growth factor
PFA: Paraformaldehyde
OCT: Optimum cutting temperature
SEM: Scanning electron microscope
PBS: Phosphate-buffered saline
CCK-8: Cell Count-8 kit
H&E: Hematoxylin and eosin
DAPI: 4′,6-diamidino-2-phenylindole
RTCA: Real-time cell analyzer
USCs-CM: USCs-conditioned medium
LSGS: Low serum growth supplementation
USCs-M: USCs-medium
BMSCs: Bone marrow-derived mesenchymal stem cells

## References

[1] Moise A, Constantinescu I, Serbanescu B, Gingu CV, Zamfirescu DG, Lascar I (2011). Hand transplant:a challenge in immunological management of patients. J Med Life.

[2] Hautz T, Zelger B, Grahammer J, Krapf C, Amberger A, Brandacher G, Landin L, Müller H, Schön M, Cavadas P (2010). Molecular markers and targeted therapy of skin rejection in composite tissue allotransplantation. Am J Transplant.

[3] Kanitakis J, Petruzzo P, Jullien D, Badet L, Dezza MC, Claudy A, Lanzetta M, Hakim N, Owen E, Dubernard J-M (2005). Pathological score for the evaluation of allograft rejection in human hand (composite tissue) allotransplantation. Eur J Dermatol.

[4] Catalano E, Cochis A, Varoni E, Rimondini L, Azzimonti B (2013). Tissue-engineered skin substitutes: an overview. J Artif Organs.

[5] Pajardi G, Rapisarda V, Somalvico F, Scotti A, Russo GL, Ciancio F, Sgrò A, Nebuloni M, Allevi R, Torre ML: **Skin substitutes based on allogenic fibroblasts or keratinocytes for chronic wounds not responding to conventional therapy: a retrospective observational study.** *Int Wound J* 2014, in press. 10.1111/iwj.12223 PMC7950180 24517418

[6] Boa O, Cloutier CB, Genest H, Labbé R, Rodrigue B, Soucy J, Roy M, Arsenault F, Ospina CE, Dubé N (2013). Prospective study on the treatment of lower-extremity chronic venous and mixed ulcers using tissue-engineered skin substitute made by the self-assembly approach. Adv Skin Wound Care.

[7] Rodrigues C, de Assis AM, Moura DJ, Halmenschlager G, Saffi J, Xavier LL, da Cruz Fernandes M, Wink MR (2014). New therapy of skin repair combining adipose-derived mesenchymal stem cells with sodium carboxymethylcellulose scaffold in a Pre-clinical Rat model. PLoS One.

[8] Machula H, Ensley B, Kellar R (2014). Electrospun tropoelastin for delivery of therapeutic adipose-derived stem cells to full-thickness dermal wounds. Adv Wound Care.

[9] Lang R, Liu G, Shi Y, Bharadwaj S, Leng X, Zhou X, Liu H, Atala A, Zhang Y (2013). Self-renewal and differentiation capacity of urine-derived stem cells after urine preservation for 24 hours. PLoS One.

[10] Zhang Y, McNeill E, Tian H, Soker S, Andersson K-E, Yoo JJ, Atala A (2008). Urine derived cells are a potential source for urological tissue reconstruction. J Urol.

[11] Bodin A, Bharadwaj S, Wu S, Gatenholm P, Atala A, Zhang Y (2010). Tissue-engineered conduit using urine-derived stem cells seeded bacterial cellulose polymer in urinary reconstruction and diversion. Biomaterials.

[12] Bharadwaj S, Liu G, Shi Y, Wu R, Yang B, He T, Fan Y, Lu X, Zhou X, Liu H (2013). Multipotential differentiation of human urine‐derived stem cells: Potential for therapeutic applications in urology. Stem Cells.

[13] Wu S, Wang Z, Bharadwaj S, Hodges SJ, Atala A, Zhang Y (2011). Implantation of autologous urine derived stem cells expressing vascular endothelial growth factor for potential use in genitourinary reconstruction. J Urol.

[14] Guan J-J, Niu X, Gong F-X, Hu B, Guo S-C, Lou Y-L, Zhang C-Q, Deng Z-F, Wang Y (2014). Biological characteristics of human-urine-derived stem cells: potential for cell-based therapy in neurology. Tissue Eng Part A.

[15] Chai C, Leong KW (2007). Biomaterials approach to expand and direct differentiation of stem cells. Mol Ther.

[16] Li WJ, Laurencin CT, Caterson EJ, Tuan RS, Ko FK (2002). Electrospun nanofibrous structure: a novel scaffold for tissue engineering. J Biomed Mater Res.

[17] Li D, Xia Y (2004). Electrospinning of nanofibers: reinventing the wheel?. Adv Mater.

[18] Nie H, He A, Jia B, Wang F, Jiang Q, Han CC (2010). A novel carrier of radionuclide based on surface modified poly (lactide- < i > co</i > −glycolide) nanofibrous membrane. Polymer.

[19] Greiner A, Wendorff JH (2007). Electrospinning: a fascinating method for the preparation of ultrathin fibers. Angew Chem Int Ed.

[20] Alvarez-Perez MA, Guarino V, Cirillo V, Ambrosio L (2010). Influence of gelatin cues in PCL electrospun membranes on nerve outgrowth. Biomacromolecules.

[21] Zhang Y, Venugopal J, Huang Z-M, Lim C, Ramakrishna S (2005). Characterization of the surface biocompatibility of the electrospun PCL-collagen nanofibers using fibroblasts. Biomacromolecules.

[22] Zhou Z, Zhou Y, Chen Y, Nie H, Wang Y, Li F, Zheng Y (2011). Bilayer porous scaffold based on poly-(ɛ-caprolactone) nanofibrous membrane and gelatin sponge for favoring cell proliferation. Appl Surf Sci.

[23] Zhang Y, Ouyang H, Lim CT, Ramakrishna S, Huang ZM (2005). Electrospinning of gelatin fibers and gelatin/PCL composite fibrous scaffolds. J Biomed Mater Res B Appl Biomater.

[24] Hartman O, Zhang C, Adams EL, Farach-Carson MC, Petrelli NJ, Chase BD, Rabolt JF (2010). Biofunctionalization of electrospun PCL-based scaffolds with perlecan domain IV peptide to create a 3-D pharmacokinetic cancer model. Biomaterials.

[25] Hajiali H, Shahgasempour S, Naimi-Jamal MR, Peirovi H (2011). Electrospun PGA/gelatin nanofibrous scaffolds and their potential application in vascular tissue engineering. Int J Nanomedicine.

[26] Chong E, Phan T, Lim I, Zhang Y, Bay B, Ramakrishna S, Lim C (2007). Evaluation of electrospun PCL/gelatin nanofibrous scaffold for wound healing and layered dermal reconstitution. Acta Biomater.

[27] Ghasemi-Mobarakeh L, Prabhakaran MP, Morshed M, Nasr-Esfahani M-H, Ramakrishna S (2008). Electrospun poly(ɛ-caprolactone)/gelatin nanofibrous scaffolds for nerve tissue engineering. Biomaterials.

[28] Revi D, Paul W, Anilkumar T, Sharma CP: **Chitosan scaffold co‐cultured with keratinocyte and fibroblast heals full thickness skin wounds in rabbit.** *J Biomed Mater Res A* 2013, in press. 10.1002/jbma.35003 24133040

[29] Jacobi J, Jang JJ, Sundram U, Dayoub H, Fajardo LF, Cooke JP (2002). Nicotine accelerates angiogenesis and wound healing in genetically diabetic mice. Am J Pathol.

[30] Galeano M, Altavilla D, Cucinotta D, Russo GT, Calò M, Bitto A, Marini H, Marini R, Adamo EB, Seminara P (2004). Recombinant human erythropoietin stimulates angiogenesis and wound healing in the genetically diabetic mouse. Diabetes.

[31] Yoon Y-s, Murayama T, Gravereaux E, Tkebuchava T, Silver M, Curry C, Wecker A, Kirchmair R, Hu CS, Kearney M (2003). VEGF-C gene therapy augments postnatal lymphangiogenesis and ameliorates secondary lymphedema. J Clin Invest.

[32] Hamano Y, Zeisberg M, Sugimoto H, Lively JC, Maeshima Y, Yang C, Hynes RO, Werb Z, Sudhakar A, Kalluri R (2003). Physiological levels of tumstatin, a fragment of collagen IV α3 chain, are generated by MMP-9 proteolysis and suppress angiogenesis via αVβ3 integrin. Cancer Cell.

[33] Jurmeister S, Baumann M, Balwierz A, Keklikoglou I, Ward A, Uhlmann S, Zhang JD, Wiemann S, Sahin Ö (2012). MicroRNA-200c represses migration and invasion of breast cancer cells by targeting actin-regulatory proteins FHOD1 and PPM1F. Mol Cell Biol.

[34] Pang H, Flinn R, Patsialou A, Wyckoff J, Roussos ET, Wu H, Pozzuto M, Goswami S, Condeelis JS, Bresnick AR (2009). Differential enhancement of breast cancer cell motility and metastasis by helical and kinase domain mutations of class IA phosphoinositide 3-kinase. Cancer Res.

[35] Martin P (1997). Wound healing–aiming for perfect skin regeneration. Science.

[36] Liang X, Boppart SA (2010). Biomechanical properties of in vivo human skin from dynamic optical coherence elastography. Biomed Eng IEEE Trans on.

[37] Falanga V (2005). Wound healing and its impairment in the diabetic foot. Lancet.

[38] Epstein FH, Singer AJ, Clark RA (1999). Cutaneous wound healing. New Engl J Med.

[39] Kim JW, Lee JH, Lyoo YS, Jung DI, Park HM (2013). The effects of topical mesenchymal stem cell transplantation in canine experimental cutaneous wounds. Vet Dermatol.

[40] Wu Y, Chen L, Scott PG, Tredget EE (2007). Mesenchymal stem cells enhance wound healing through differentiation and angiogenesis. Stem Cells.

[41] Bharadwaj S, Liu G, Shi Y, Markert C, Andersson K-E, Atala A, Zhang Y (2011). Characterization of urine-derived stem cells obtained from upper urinary tract for use in cell-based urological tissue engineering. Tissue Eng Part A.

[42] Wu S, Liu Y, Bharadwaj S, Atala A, Zhang Y (2011). Human urine-derived stem cells seeded in a modified 3D porous small intestinal submucosa scaffold for urethral tissue engineering. Biomaterials.

[43] Hofmann M, Wollert KC, Meyer GP, Menke A, Arseniev L, Hertenstein B, Ganser A, Knapp WH, Drexler H (2005). Monitoring of bone marrow cell homing into the infarcted human myocardium. Circulation.

[44] Arnold F, West DC (1991). Angiogenesis in wound healing. Pharmacol Ther.

[45] Chen L, Tredget EE, Wu PY, Wu Y (2008). Paracrine factors of mesenchymal stem cells recruit macrophages and endothelial lineage cells and enhance wound healing. PLoS One.

[46] Sachewsky N, Hunt J, Cooke MJ, Azimi A, Zarin T, Miu C, Shoichet MS, Morshead CM (2014). Cyclosporin A enhances neural precursor cell survival in mice through a calcineurin-independent pathway. Dis Model Mech.

